# *Abies Concolor* Seeds and Cones as New Source of Essential Oils—Composition and Biological Activity

**DOI:** 10.3390/molecules22111880

**Published:** 2017-11-02

**Authors:** Anna Wajs-Bonikowska, Łukasz Szoka, Ewa Karna, Anna Wiktorowska-Owczarek, Monika Sienkiewicz

**Affiliations:** 1 Institute of General Food Chemistry, Biotechnology and Food Science, Lodz University of Technology, Stefanowskiego St. 4/10, 90-924 Łódź, Poland; 2 Department of Medicinal Chemistry, Medical University of Bialystok, Kilińskiego St. 1, 15-089 Białystok, Poland; lukasz.szoka@umb.edu.pl (Ł.S.); ewa.karna@umb.edu.pl (E.K.); 3 Pharmacology and Toxicology Department, Medical University of Lodz, Żeligowskiego St. 7/9, 90-752 Łódź, Poland; anna.wiktorowska-owczarek@umed.lodz.pl; 4 Department of Allergology and Respiratory Rehabilitation, Medical University of Lodz, Hallera Sq.1, 90-549 Łódź, Poland; monika.sienkiewicz@umed.lodz.pl

**Keywords:** *Abies concolor*, fir, essential oil, seed, cone, antimicrobial activity, cytotoxicity

## Abstract

The chemical composition, including the enantiomeric excess of the main terpenes, of essential oils from seeds and cones of *Abies concolor* was studied by chromatographic (GC) and spectroscopic methods (mass spectrometry, nuclear magnetic resonance), leading to the determination of 98 compounds. Essential oils were mainly composed of monoterpene hydrocarbons. The dominant volatiles of seed essential oil were: limonene (47 g/100 g, almost pure levorotary form) and α-pinene (40 g/100 g), while α-pinene (58 g/100 g), sabinene (11 g/100 g), and β-pinene (4.5 g/100 g) were the predominant components of the cone oil. The seed and cone essential oils exhibited mild antibacterial activity, and the MIC ranged from 26 to 30 μL/mL against all of the tested bacterial standard strains: *Staphylococcus aureus*, *Enterococcus faecalis*, *Enterococcus faecium*, *Escherichia coli*, and *Klebsiella pneumoniae*. The cytotoxic studies have demonstrated that tested essential oils were cytotoxic to human skin fibroblasts and human microvascular endothelial cells at concentrations much lower than the MIC. The essential oils from *A. concolor* seeds and cones had no toxic effect on human skin fibroblasts and human microvascular endothelial cells, when added to the cells at a low concentration (0–0.075 μL/mL) and (0–1.0 μL/mL), respectively, and cultured for 24 h.

## 1. Introduction

*Abies concolor*, commonly known as the white fir, is a species native to the mountains of western North America (Sierra Nevada Mountains of southern Oregon and California, as well the southern Rocky Mountains). Variation between the two regions of *A. concolor* is sufficient to commonly recognize two varieties, i.e., var. *Iowiana* (California white fir) and var. *concolor* (Rocky Mountain white fir). Only the Rocky Mountain form of Concolor fir is suited for planting in other northern hemisphere regions [1,2]. White fir was discovered by William Lobb on his expedition to California in the middle of the 19th century. This tree naturally occurs at elevations of 900–3400 m and grows to 25–60 m tall. The cones are 6–12 cm long and 4–4.5 cm broad, green or purple, with about 100–150 scales. The winged seeds are released when the cones disintegrate at maturity about six months after pollination. In Poland it is usually in September. *Abies concolor* generally lives from 300 to 400 years. Some trees have been known to live to even over 500 years. White fir is a preferred construction species because of its nail-holding ability, lightness in weight, and resistance to split, twist, and pitch. It is straight-grained, non-resinous, fine-textured, stiff, and strong. This fir species is widely planted in northern hemisphere as an ornamental tree in parks and larger gardens, and also cultivated as a Christmas tree [3].

*Abies concolor* had, and still has, many uses in folk medicine. For example, Indians used an infusion of the foliage, which can help to treat rheumatism and pulmonary troubles. Resin decoction was used for venereal diseases. The resin has also been known to be used by early New Mexico natives to fill decayed teeth. *Abies concolor* needle is still used by many Native Americans as a tea [4]. Extracts of the bark of *Abies concolor* have shown antitumor activity against adenocarcinoma [5]. To the best of our knowledge, no other biological screening of any *A. concolor* isolates (including essential oils) have been done. Due to essential oils, antibacterial, antifungal, and insecticidal activities, these plant isolates have been intensely tested and applied in the fields of pharmacology, medical and clinical microbiology, and food preservation. As it was found in many current scientific literature, the essential oils have also been shown to possess cytotoxic effects. The remarkable effectiveness exhibited by the essential oils may be interesting for example in the cosmetic industry or food processing, which, as is known, is increasingly directed to the use of so called green chemistry that is able to safeguard the high quality of products and the environment [6].

*Abies concolor* is a very poorly investigated fir species. In contrast to a few manuscripts that reported needle volatiles [7,8], variability of needle terpenes during seasonal changes [9,10], as well of needle essential oils of firs growing in urban environment [11], and two papers dealing with seed fatty acids of white fir [12,13], no attention has been paid to intact seed or cone essential oils, both with respect to their chemistry and biological functions.

Thus, the aim of our study was to fill the gap in the composition and biological activity of white fir seed and cone essential oils.

## 2. Results and Discussion

### 2.1. Chemistry of Essential Oils

The yield of cone essential oil ranged from 0.40% to 0.47% (average—0.44%). The yield of seed essential oil was twelve times higher than that of cone scales and was on the level of 5.43% (ranging from 5.38% to 5.54%). Seed oil was also characterized by very attractive forestry, resinous, fresh, citrus-like scent, opposite to less attractive, resinous, soil-like scent of cone oil, caused *inter alia*, probably by a bigger amount of high boiling volatiles, like the diterpenes that are detected in cone oil. The yield of *A. concolor* seed oil is slightly higher than the average yield of oil from *A. koreana* seeds (4.9%) [14], while being much lower in comparison with *Abies alba* seed oils (7.4–14.3%) [15,16]. Chromatographic and spectroscopic methods allowed us to identify 98 compounds in the examined essential oils, which constituted 92.3 g/100 g and 96.8 g/100 g of cone and seed oils, respectively. The structure of 13 of white fir volatiles were also confirmed by ^1^H-NMR spectroscopy. The identified compounds of seed and cone essential oils are shown in Table 1. The main terpene group detected in both seed and cone essential oils were monoterpene hydrocarbons, constituting 93 g/100 g and 79 g/100 g, respectively, like in the *Abies concolor* needle essential oils, where the concentration of this terpene group was about 90% [7,8,9,10]. Monoterpene hydrocarbons were also a predominant chemical group in the oils that were isolated from seeds and cones of both *Abies alba* [15,16] and *A. koreana* [14,15,16], The dominant monoterpene hydrocarbons and at the same time the main constituents of examined seed essential oil were limonene (47 g/100 g) and α-pinene (40 g/100 g). It is important that the levorotatory form of limonene in the seed oil was in great majority (≥97%). This valuable flavor compound, and exactly its levorotatory enantiomer, was also the main component of silver [15,16] and Korean fir seed essential oils [14,15,16,17]. Our results indicated that α-pinene (58 g/100 g), sabinene (11 g/100 g), and β-pinene (4.5 g/100 g) were the predominant components of the *Abies concolor* cone oil. Despite the fact that the previously analysed needle essential oils were isolated from a different population of *A. concolor*, still the main constituent was β-pinene constituting up to 52% of oils [9,10,11]. Limonene (up to 23%), camphene (up to 17%), β-phellandrene (up to 14%) and α-pinene (up to 10%) were also detected in the needle essential oils as the dominant volatiles [7,8,9,10,11].

The second most abundant group characterizing the examined seed oil were sesquiterpene hydrocarbons (1.9 g/100 g), among which γ-cadinene (0.97 g/100 g), α-muurolene (0.20 g/100 g), and α-copaene (0.18 g/100 g) were the main compounds. This terpene group constituted from 5% to even 10% of the needle oil. Among them, δ- and γ-cadinene, valencene, and (*E*)-β-caryophyllene were the major in needle oils [7,8,9,10].

Opposite to the seed oil, the second main and most numerous group in the examined cone oil were oxygenated derivatives of monoterpenes (9.2 g/100 g). In this group predominant was terpinen-4-ol (1.9 g/100 g), myrtenol and verbenol (2.2 g/100 g), *trans*-pinocarveol (1.4 g/100 g), as well as *trans*-verbenol (1.2 g/100 g). It is worth mentioning that the main oxygenated monoterpenes in the needle oil were bornyl acetate and α-terpineol, which dependently on population origin constituted even 20% and 7% of essential oil, respectively [7,8,9,10]. Bornyl acetate, which has been found in the tested *A. concolor* seed and cone oil at a very low level (0.33 and 0.31 g/100 g, respectively), had been found previously as the main oxygenated monoterpene, as well as one of the main volatiles, both in *A. koreana* seed (up to 18.6%) and cone (up to 12.4%) oils [14,16], however the content of this compound depended strongly on the tree specimen. Interestingly, bornyl acetate constituted only traces in the *Abies alba* seed and cone essential oils [15,16].

Diterpene hydrocarbons (1.3 g/100 g) and their oxygenated derivatives (1.5 g/100 g) occurred only in the *A. concolor* cone essential oil. 18-Norabieta-8,11,13-triene, abieta-8(14),9(11),13-triene, dehydroabietal, and pimara-8(14),15-diene were the main diterpenes. Seed essential oil is characterized by traces of only one diterpene, i.e., abieta-8(14),9(11),13-triene. High boiling diterpenes also occurred at a very low level, both in the white fir needle oils [7,8,9,10,11] and in the silver and Korean fir seed and cone essential oils (<0.3%) [14,15,16,17].

Among volatile terpenes, the oxygenated derivatives of sesquiterpenes were detected in the analyzed white fir at the lowest level in both seed (0.21 g/100 g) and cone oils (0.04 g/100 g). This group consisted mainly of τ-cadinol and τ-muurol. However, this terpene group was detected in the *A. concolor* needle oil at a much higher level, up to 6.4% [7,8,9,10]. Higher than in white fir oils, the amount of oxygenated sesquiterpenes has been found previously in the silver fir seed cone oils (up to 1.5%) and Korean fir seed and cone oils (up to 1.6%). Intermedeol and selin-6-en-ol were the main one [14,15,16,17].

As it is shown in Table 1, the quantitative as well as qualitative compositions of seed and cone oil of white fir are different. Despite the fact that all of the main compounds, i.e., monoterpene hydrocarbons, were identified in seed and cone essential oils, many terpenes occurred only in one of the examined oils. Finally, among the 98 compounds identified in both white fir oils, 61 volatiles were detected in the seed oil, while 70 terpenes in the cone oil. As can be seen, the most significant differences between both oils is the content of limonene, which constituted 47.15 g/100 g in the seed oil, while cone oil contained only 2.71 g/100 g of this monoterpene. What is more, sabinene is one of the main compounds in the cone oil (10.73 g/100 g), but it constituted only 0.23 g/100 g of the seed oil. Another determinant that distinguishes cone oil from seed oil is a higher content of oxygenated derivatives of monoterpenes, which constituted 9.23 g/100 g and 0.71 g/100 g, respectively. Among this group, *trans*-pinocarveol, *trans*-verbenol, myrthenol and verbenol, α-terpineol, cryptone and α-campholenal occurred in significantly higher concentrations in comparison with *A. concolor* seed oil. Probably due to the ligneous structure of cone scales and ability to produce resin the cone oil contained also a higher concentration of diterpenes, 2.82 g/100 g, while seed oil contained only traces of these chemicals.

Natural essential oil that contains chiral molecules often shows the presence of a predominant enantiomer for each pair, and the enantiomeric ratio is therefore a value that characterizes the genuineness of an oil [18]. The enantiomeric excess of limonene, α- and β-pinene as well as camphene, is listed in Table 2. The levorotatory form of the main monoterpene hydrocarbons constituting the seed and cone oils of the investigated fir is largely predominant over the dextrorotatory form. This dependence was also observed for the above mentioned monoterpene hydrocarbons, which are the components of essential oils from the seeds and cones of *A. alba* and *A. koreana* [14,17]. Enantiomeric excess of (–) form of terpenes tested in that study is usually between 59 and 94%, except for β-pinene in the white fir seed oil, where enantiomeric excess of dextrorotatory form is 79.8%.

### 2.2. Biological Activities of Essential Oils

The Gram-positive and Gram-negative bacteria are the cause of severe infections in humans, and due to their ability to survive in harsh conditions they may cause hospital infections. The use of natural products would be a helpful way to reduce the risk of growth of bacterial resistance to antibiotics. There is a need to develop alternative antimicrobial products from plants to support antibacterial therapy in the treatment of infectious diseases. Essential oils exhibit antimicrobial activity and affect processes occurring in cells that could alter human cell viability. This makes it necessary to confront the concentration that would have a cytotoxic effect on human cells with MIC.

The antibacterial activity of the seed and cone essential oils of the examined white fir was evaluated against *Staphylococcus aureus*, *Enterococcus faecalis*, *Enterococcus faecium*, *Escherichia coli*, and *Klebsiella pneumoniae* reference strains (Table 3). When compared to those of thymol, which was used as a positive control, the tested oils showed rather low antimicrobial properties. Despite the fact that the compositions of seed and cone essential oils are different, the results of the antimicrobial screening showed that the *A. concolor* seed oil exhibited almost identical antibacterial properties to cone oil (minimal inhibitory concentrations was in the range of 26 to 30 μL/mL). It is due to a high and similar content of monoterpene hydrocarbons in both essential oils (93 g/100 g of seed oil, 79 g/100 g of cone oil), which in contrast to oxygenated terpenes show the lowest antibacterial activity, even hydrocarbons tend to be relatively inactive. This is caused by their structure that limits hydrogen bound capacity and water solubility, and as a result, limits their diffusion through the medium. Limonene, α-pinene, β-pinene, sabinene, and β-myrcene, which are the predominant oil components showed a very low or no antimicrobial activity. On the other hand, more polar molecules like alcohols, aldehydes or ketones, ethers, or oxides, are active, but with differing specificity and levels of activity, which is related to the present functional group, but also associated with hydrogen-bounding parameters in all cases [18,19]. Surprisingly, we did not notice the differences of antimicrobial activity between seed and cone oils of white fir, although the concentration of oxygenated derivatives of mono-, sesqui- and diterpenes in cone essential oils was ten times higher (10% of all volatiles) than in seed oil (0.9% of all oil compounds). Thymol, was used as a positive control, and obviously was much more effective against tested bacteria strains than the *A. concolor* oils. It is well known that the essential oil constituents confer antimicrobial activity by damaging the cell membrane and wall, leading to cell lysis, leakage of cell contents, as well the inhibition of proton motive force [6].

According to the literature, the essential oils isolated from *A. alba* and *A. koreana* seeds and cones showed a stronger antimicrobial activity against *S. aureus*, *E. faecalis*, and *E. faecium* (MIC: 10–23 μL/mL) [16] than those isolated from *A. concolor* seeds and cones (MIC: 26–30 μL/mL). It was probably caused by a higher concentration of oxygenated derivatives of terpenes in silver and Korean fir oils. Both seed and cone essential oils of white fir exhibited similar antibacterial properties against *E. coli* and *K. pneumoniae* as those of silver fir and Korean fir seed and cone oils (MIC: 25.5–34 μL/mL) described in the literature [16].

**Figure 1:**
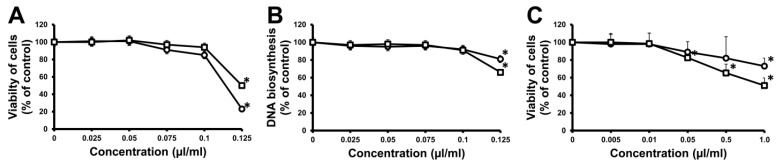
Viability (**A**) and DNA synthesis (**B**) of human skin fibroblasts and viability (**C**) of human microvascular endothelial cells (HMEC-1) incubated for 24 h with various concentrations of essential oils derived from *Abies concolor* seeds (□) and cones (○). Cell viability and DNA synthesis are expressed as percentage when compared to untreated control cells and given as mean ± standard deviation of three independent experiments done in triplicates. Significant differences (*p* ≤ 0.05) are indicated by *.

Two essential oils (*Abies concolor* seeds and *A. concolor* cones) were examined in a cell viability test on human fibroblasts, using 3-(4,5-dimethylthiazol-2-yl)-2,5-diphenyl-2*H*-tetrazolium bromide (MTT). Thymol was used as a positive control. The results of cytotoxicity of the tested oils are shown in Figure 1. It was observed that at a concentration of 0–0.075 μL/mL the oils were rather safe. Their IC_50_ value fluctuated around 0.110 μL/mL (*Abies concolor* cones) and 0.125 μL/mL (*Abies concolor* seeds). The influence of oils on normal cells was weak when compared to thymol, whose IC_50_ fluctuated around 75 μg/mL, which corresponds to ca. 0.075 μL/mL (due to the melting point of thymol which was weighted). The effect of oils was confirmed by DNA synthesis. The oil from *Abies concolor* cones at a concentration of 0.110 μL/mL decreased DNA synthesis to 86% of the control value. The oil from *Abies concolor* seeds at a concentration of 0.125 μL/mL decreased DNA synthesis to 66% of the control value. Thymol used as a positive control reduced DNA synthesis to 20% and 15% of the control value, respectively. The influence of white fir essential oils was also examined in a cell viability test on human microvascular endothelial cells (HMEC-1), using MTT. The studies demonstrated that the tested essential oils were cytotoxic to HMEC-1 at concentrations lower than the MIC. The oil from *A. concolor* cones was less toxic than the seed oil. The IC_50_ of seed and cone essential oil was 1.0 μL/mL and 1.38 μL/mL, respectively.

## 3. Experimental

### 3.1. Plant Material

Cones with ripe seeds were collected from about 40 years old tree of *Abies concolor*, *var. concolor*, growing in the Polish Botanical Garden in the centre of Poland (Łódź). The fresh material (cones with seeds) was hand-picked from the top of the tree, in September 2012 and was identified by Łódź Botanical Garden specialist. The samples were stored in tight plastic bags in a freezer (−24 °C) until they were needed. Before hydrodistillation, the seeds were separated from cone scales and ground in a mill. The voucher specimen of plant material was deposited in the Institute of General Food Chemistry, Lodz, Poland.

### 3.2. Essential Oil Isolation

Before hydrodistillation the fir seeds were crushed using simple mill. Essential oils from such crushed seeds and separately from cone scales were isolated by hydrodistillation for 4 h using a Clevenger-type glass apparatus. After that time, the amount of essential oils in apparatus did not rise, thus we choose 4 h as an appropriate time of seed essential oil isolation. Hydrodistillation was performed 14 times for cone scales and three times for seeds. The number of distillation was caused by huge amount of cone scales and less weight of seeds. Each hydrodistillation was conducted using 100–200 g of plant material.

### 3.3. Chromatographic Analysis

Essential oils, its enantiomers, were analysed by GC-MS-FID. The analyses were performed on a Trace GC Ultra coupled with DSQII mass spectrometer (Thermo Electron, Waltham, MA, USA). A simultaneous GC-FID and MS analysis was performed using a MS-FID splitter (SGE Analytical Science, Ringwood Victoria, Australia). Mass range was 33–550 amu, ion source-heating: 200 °C, ionization energy: 70 eV. One microliter of essential oil solution (80% *v*/*v*) diluted in pentane:diethyl ether was injected in split mode at split ratios (50:1). Operating conditions: capillary column Rtx-1 MS (60 m × 0.25 mm i.d., film thickness 0.25 μm), and temperature program: 50 (3 min)–300 °C (30 min) at 4 °C/min. Injector and detector temperatures were 280 °C and 300 °C, respectively. Carrier gas was helium (constant pressure: 300 kPa). Resolution of (−) and (+) enantiomers of limonene, α- and β-pinene, camphene was performed on a Chirasil-Dex CB column (Agilent, Santa Clara, CA, USA) having the following dimensions: 30 m × 0.25 mm i.d., 0.25 μm df. Temperature program: 50 (3 min)–220 °C (30 min) at 4 °C/min. Injector and detector temperatures were 240 °C and 250 °C, respectively. Carrier gas was nitrogen at the flow rate of 1.0 mL/min.

For the measurement of response factors, compounds determined in the volatile fraction of the oil were grouped, based on their moieties, into chemical classes (hydrocarbons, aldehydes, etc.); each class was sub-divided depending on the comprehensive structure of the compounds e.g., monoterpene, sesquiterpene, etc.). Standard compounds, each representing the chemical classes determined, were selected among those available in the laboratory. For a high level of reliability, when possible, more than one standard compound per class was considered.

### 3.4. Identification of Compounds

The identification of compounds was based on a comparison of MS spectra with computer mass library NIST 2012, Wiley Registry of Mass Spectral Data 8th edition, and MassFinder 4.1 along with the relative retention indices (RI, non-polar column). Identification was also based on the comparison of ^1^H-NMR spectra with literature data or a homemade NMR database. The identification of limonene, camphene, α-, and β-pinene enantiomers was based on (−)- and (+)-standards of these terpenes.

### 3.5. Isolation of Components

To isolate the volatiles of interest, the seed oil (34 g) was subjected to fractional distillation under reduced pressure. The residue, deprived of monoterpene hydrocarbons (1.32 g) was flash-chromatographed (FC) on silica gel 60 (0.040–0.063 mm, Merck, EM Science, NJ, USA) with hexane and increasing amounts of diethyl ether. The separation was monitored by TLC and GC-MS. Fourteen fractions (1–14) were obtained and analysed by GC-MS-FID. Structures of 13 volatiles from the following fractions were confirmed using ^1^H-NMR spectroscopy: 1: (157 mg) α-copaene (33%), α-cubebene (37%); 2: (690 mg) γ-cadinene (26%), δ-cadinene (31%); 7: (526 mg) bornyl acetate (82%); 9: (131 mg) terpinen-4-ol (24%), cubenol (39%); 10: (34 mg) τ-cadinol (51%); 11: (129 mg) 4-*epi*-cubebol (33%); 12: (176 mg) α-cadinol (27%), *cis*-verbenol (24%), verbenon (23%); and, 14: (33 mg) *trans*-calamenene-10-ol (37%).

### 3.6. Essential Oil Constituents Quantification

Quantification of the seed and cone essential oil components was carried out based on the measurement of response factors for all of the chemical groups. The application of this analytical procedure allowed the accurate determination of the concentration (g/100 g) of all the tested components to be made. For the calculation of response factors, the standard compounds mentioned below were spiked with nonane as internal standard and injected consecutively for three runs. Once calculated, response factors were used for the absolute quantification of volatiles, based on the commonly known formula. The application of this analytical procedure allowed for the determination of the concentration (g/100 g) of all the 98 compounds identified. For the measurement of response factors, the following chemicals were used: α-pinene, β-pinene, camphene, limonene for monoterpene hydrocarbons (*R*_f_ = 0.99 ± 0.050); (*E*)-caryophyllene and germacrene D, for sesquiterpene hydrocarbons (*R*_f_ = 0.99 ± 0.019); *p*-cymene, for aromatic hydrocarbons (*R*_f_ = 0.99 ± 0.004); octanal, decanal and citral, for aldehydes (*R*_f_ = 1.28 ± 0.050); benzaldehyde, for aromatic aldehydes (*R*_f_ = 1.24 ± 0.042); heptan-2-one and pulegone, for ketones (*R*_f_ = 1.34 ± 0.053); citronellol, terpinen-4-ol, geraniol, *cis*-hex-3-en-1-ol, borneol, eugenol, thymol, farnesol and carotol for alcohols (*R*_f_ = 1.29 ± 0.071), buthyl acetate, neryl acetate, benzyl benzoate for esters (*R*_f_ = 1.55 ± 0.045); caryophyllene oxide and safrol, for oxides (*R*_f_ = 1.53 ± 0.002); and, ethers (*R*_f_ = 1.46 ± 0.009). Response factors for diterpene hydrocarbons were measured as an average of *R*_f_s for monoterpene and sesquiterpene hydrocarbons (*R*_f_ = 0.99).

### 3.7. Antimicrobial Activity

The standard bacterial strains, i.e., the Gram-positive strains *Staphylococcus aureus* ATCC 43300, *Enterococcus faecalis* ATCC 51299 (VanB), and *Enterococcus faecium* VA sensitive ATCC 35667 and the Gram-negative strains *Escherichia coli* ATCC 25922 and *Klebsiella pneumoniae* ATCC 700603 were obtained from the collection of the Medical and Sanitary Microbiology Department, Medical University of Łódź (Łódź, Poland). The standard strains were cultivated in Columbia agar medium and were incubated at 37 °C for 24 h in aerobic conditions. Bacterial suspensions with an optical density of 0.5 McFarland scale were prepared and analyzed with a BioMerieux densitometer (BioMerieux, Hamburg, Germany). The antibacterial activity of the oils was investigated with the broth micro-dilution method [20,21]. The essential oils and thymol (used as a positive control) were diluted in EtOH. These solutions were mixed with 100 μL of Mueller-Hinton broth to obtain concentrations from 11.5 to 32.5 μL/mL. The mixtures were then transferred to 96-well microliter plates. An inoculum (10 μL) per well was added to the broth with various oil concentrations. As a control of strain growth, one well was filled with broth without oils or thymol. The minimal inhibitory concentration (MIC) was determined as the lowest concentration of oil or thymol, which inhibited the visible growth of bacteria after 24 h of incubation at 37 °C under aerobic conditions. The control media containing only EtOH at concentrations that were used for the dilutions of the oils did not inhibit the growth of the standard bacterial strains.

### 3.8. Cell Culture

HMEC-1 (human microvascular endothelial cells) were purchased from ATCC (Rockville, MD, USA), catalog number ATCC-CRL-10636 (depositor Centers for Disease Control, Dr. Edwin W. Ades, Atlanta). For experimentation, the cells between passages 10–31 were used. HMEC-1 cells were cultured in 25 cm^3^ flasks in MCDB 131 medium that was supplemented with 10% fetal bovine serum (Invitrogen, Carlsbad, CA, USA), 10 ng/mL epidermal growth factor, 1 μg/mL hydrocortisone and penicillin-streptomycin solution (Sigma-Aldrich Chemical Co. Ltd., St. Louis, MO, USA), in a humidified atmosphere of 95% and 5% CO_2_ at 37 °C. Cells were harvested every third day in a trypsin-EDTA solution (0.25% trypsin, 1 mM EDTA). HMEC-1 cells were cultured according to the method described in the literature [22], and the author’s own modification [23].

Normal human skin fibroblasts (CRL-1474) were obtained from the American Type Culture Collection (Manassas, VA, USA). The cells were maintained in Dulbecco’s modified Eagle medium (Gibco, Gaithersburg, MD, USA) supplemented with 10% fetal bovine serum (Gibco, Washington, DC, USA), 50 U/mL penicillin and 50 μg/mL streptomycin at 37 °C in a 5% CO_2_ incubator. Cells were counted in a hemocytometer and cultured at 1 × 10^5^ cells per well in 2 mL of growth medium in 96 or 24 well plates (Costar, Washington, DC, USA). Cells reached confluence at day 6, and in most cases such cells were used for assays. The essential oils and thymol were diluted in ethanol. Ethanol was added also to the control. The final ethanol concentration did not exceed 0.1% throughout the study.

### 3.9. Cell-Viability Assay

The assay was performed according to the literature [24] using 3-(4,5-dimethylthiazol-2-yl)-2,5-diphenyl-2*H*-tetrazolium bromide (MTT; Sigma-Aldrich Chemical Co. Ltd.; St. Louis, MO, USA). HMEC-1 (human microvascular endothelial cells) cells and human skin fibroblasts were cultured for 24 h with various concentrations: of essential oils or thymol and without tested oils or thymol (control groups) in 96-well plates. After incubation, 50 μL MTT (1 mg/mL, Sigma) was added and the plates were incubated at 37 °C for 3 h. At the end of the experiment, the cells were exposed to 100 μL dimethyl sulphoxide, which enabled the release of the blue reaction product: formazan. The absorbance at 570 nm was read on a microplate reader and the results were expressed as a percentage of the absorbance measured in control cells. The results were submitted to statistical analysis using ANOVA, and post-hoc comparisons were performed using the Student-Newman-Keuls test. The normal distribution of parameters was checked by means of the Shapiro-Wilk test. If the data was not normally distributed or the values of the variance (test *F*) were different, ANOVA with Kruscal-Wallis and Mann-Whitney’s *U* test were used. All of the parameters were considered significantly different if *p* < 0.05. The statistical data analysis was performed using Statgraphics 5.0 plus software (version 5.0 manuals, STSC Inc., Rockville, MD, USA).

### 3.10. DNA Biosynthesis Assay

To examine the effect of essential oils from *Abies concolor* cones and *A. concolor* seeds on fibroblast proliferation, the cells were plated in 24-well tissue culture dishes at 1 × 10^5^ cells/well with 1 mL of growth medium. After 24 h [(1.6 ± 0.1) × 10^5^ cells/well], the plates were incubated with various concentrations of two essential oils or thymol and 0.5 μCi of [^3^H] thymidine for 24 h at 37 °C. Cells were rinsed three times with phosphate buffered saline, solubilized with 1 mL of 0.1 M sodium hydroxide containing 1% sodium dodecyl sulfate. Scintillation liquid (9 mL) was added and radioactivity incorporated into DNA was measured in a scintillation counter [25].

## 4. Conclusions

Summarizing, the seeds of *Abies concolor* are a rich source of essential oil (5.4%) with a very pleasant forestry scent. The quantitative and qualitative compositions of examined seed and cone essential oils differ from each other, however the main volatiles belong to monoterpene group. In conclusion, our results indicate that the essential oils from *Abies concolor* seeds and cones have no toxic effect on human skin fibroblasts and human microvascular endothelial cells when added to the cells at a low concentration (0–0.075 μL/mL and 0–1.0 μL/mL, respectively) and cultured for 24 h. The inhibiting activity of essential oils isolated from *A. concolor* seeds and cones was mild, and this is certainly dependent on the presence of monoterpene hydrocarbons as a mine constituents of tested oils, but essential oils from these conifers may be a valuable addition to medicines and cosmetics with a gentle antibacterial action.

## Figures and Tables

**Table 1:** Composition of *Abies concolor* seed and cone essential oils.

		Seed Essential Oil	Cone Essential Oil	RI Exp.	Identification Method
No.	Compound	(g/100 g)	(g/100 g)		
1	Santolinatriene		0.03	914	RI, MS
2	Tricyclene	0.02	0.02	918	RI, MS
3	α-Thujene	0.01	0.55	923	RI, MS
4	**α-Pinene**	**40.22**	**58.31**	933	RI, MS
5	Camphene	0.3	0.41	941	RI, MS
6	Thuja-2,4-(10)-diene	0.02	0.28	944	RI, MS
7	**Sabinene**	0.23	**10.73**	963	RI, MS
8	**β-Pinene**	**3.54**	**4.45**	967	RI, MS
9	1,8-Dehydrocineol		0.02	979	RI, MS
10	Verbene		0.16	980	RI, MS
11	**β-Myrcene**	**1.39**		986	
12	δ-Car-2-ene	0.01	0.02	992	RI, MS
13	α-Phellandrene			999	
14	δ-Car-3-ene		0.13	1000	RI, MS
15	α-Terpinene	0.08	0.2	1009	RI, MS
16	*p*-Cymene		0.83	1008	RI, MS
17	**Limonene**	**47.15**	**2.71**	1025	RI, MS
18	β-Phellandrene		0.01	1026	RI, MS
19	γ-Terpinene	0.01		1047	RI, MS
20	trans-Sabinene hydrate	0.01	0.06	1052	RI, MS
21	Fenchon	0.02		1066	RI, MS
22	*p*-Cymenene		0.02	1066	RI, MS
23	Terpinolene	0.03	0.69	1081	RI, MS
24	*cis*-Sabinene hydrate		0.06	1083	RI, MS
25	Linallol	0.03		1090	RI, MS
26	α-Thujone		0.01	1090	RI, MS
27	α-Fenchol	0.01	0.04	1099	RI, MS
28	α-Campholenal	0.01	0.24	1104	RI, MS
29	*cis*-*p*-Menth-2-en-1-ol		0.04	1106	RI, MS
30	*cis*-Limonene oxide	0.02		1115	RI, MS
31	*cis*-*p*-Mentha-2,8-dien-1-ol	0.01		1119	RI, MS
32	Camphor	0.01	0.02	1124	RI, MS
33	***trans*-Pinocarveol**		**1.37**	1125	RI, MS
34	*cis*-Verbenol	0.02		1129	^1^H, RI, MS
35	***trans*-Verbenol**	0.03	**1.18**	1129	RI, MS
36	α-Phellandren-8-ol			1133	
37	Pinocarvon	0.03		1137	RI, MS
38	Cryptone		0.27	1149	RI, MS
39	α-Thujenal		0.07	1159	RI, MS
40	**Terpinen-4-ol**	0.03	**1.99**	1162	^1^H, RI, MS
41	Myrtenal	0.03	0.33	1169	RI, MS
42	α-Terpineol	0.03	0.28	1173	RI, MS
43	*trans*-Dihydrocarvon		0.01	1177	RI, MS
44	**Myrtenol**		**2.17**	1182	^1^H, RI, MS
45	**Verbenone**	0.01		1188	
46	*cis*-Carvotanacetone	0.01		1190	RI, MS
47	*trans*-Carveol	0.01	0.02	1199	RI, MS
48	Citronellol	0.01		1211	RI, MS
49	*cis*-Carveol		0.01	1212	RI, MS
50	Thymol methyl ether	0.02	0.01	1215	RI, MS
51	Carvon		0.01	1221	RI, MS
52	Piperitone		0.01	1223	RI, MS
53	Bornyl acetate	0.33	0.31	1267	^1^H, RI, MS
54	*trans*-Shisol		0.01	1279	RI, MS
55	Perilla aldehyde		0.01	1283	RI, MS
56	*p*-Mentha-1,4-dien-7-ol		0.01	1305	RI, MS
57	α-Cubebene	0.15	0.01	1346	^1^H, RI, MS
58	α-Copaene	0.18		1373	^1^H, RI, MS
59	α-Bourbonene		0.39	1381	RI, MS
60	β-Bourbonene	0.01		1385	RI, MS
61	(*E*)-β-Caryophyllene	0.04	0.01	1414	RI, MS
62	α-Bergamotene		0.02	1424	RI, MS
63	*trans*-β-Farnesene		0.02	1438	RI, MS
64	Cadina-3,5-diene	0.01		1447	RI, MS
65	Cadina-4,11-diene	0.01		1456	RI, MS
66	γ-Muurolen	0.17		1467	RI, MS
67	Germacrene D	0.05	0.03	1473	RI, MS
68	β-Selinene	0.02		1479	RI, MS
69	Bicyclosesquiphellandrene	0.04		1485	RI, MS
70	4-epi-Cubebol	0.02		1488	^1^H, RI, MS
71	α-Muurolene	0.2		1490	RI, MS
72	**γ-Cadinene**	**0.97**	0.01	1504	¹H, RI, MS
73	*cis*/*trans*-Calamenene	0.01		1509	RI, MS
74	Cadina-1,4-diene	0.01		1528	RI, MS
75	δ-Cadinene	0.02		1528	^1^H, RI, MS
76	(*E*)-Nerolidol	0.01		1547	RI, MS
77	Gleenol	0.01		1571	RI, MS
78	1,10-di-epi-Cubenol	0.01		1603	RI, MS
79	Selin-6-en-4-ol				
80	1-epi-Cubenol	0.02		1613	RI, MS
81	τ-Cadinol	0.13	0.02	1625	^1^H, RI, MS
82	τ-Muurolol				RI, MS
83	Cubenol			1629	^1^H, RI, MS
84	α-Cadinol	0.01	0.02	1638	^1^H, RI, MS
85	*cis*-Calemenen-10-ol	0.01		1640	RI, MS
86	*trans*-Calemenen-10-ol	0.01		1647	^1^H, RI, MS
87	Cembrene		0.01	1918	RI, MS
88	18-Norabieta-8,11,13-triene		0.42	1965	RI, MS
89	Manoyl oxide		0.08	1987	RI, MS
90	Pimara-8(14),15-diene		0.21	1998	RI, MS
91	Methyl abietate		1.10	1996	RI, MS
92	Kaur-16-ene		0.04	2029	RI, MS
93	Abieta-8(14),9(11),13-triene	0.06	0.35	2038	RI, MS
94	Abieta-8-(14),13(15)-dien		0.30	2075	RI, MS
95	Cembrol		0.02	2139	RI, MS
96	Pimara-7,15-dien-3-on		0.03	2225	RI, MS
97	Dehydroabietal		0.23	2233	RI, MS
98	Abietal		0.06	2286	RI, MS
	Monoterpene hydrocarbons	92.98	78.88		
	Oxygenated monoterpenes	0.71	9.23		
	Sesquiterpene hydrocarbons	1.89	0.49		
	Oxygenated sesquiterpenes	0.21	0.04		
	Diterpene hydrocarbons	0.06	1.32		
	Oxygenated diterpenes	-	1.50		
	Others	0.01	0.03		
	**Total identified [g/100 g]**	**95.86**	**91.87**		
	**Yield of essential oil [%]**	**5.43**	**0.44**		
	± (standard deviation)	±(0.09)	±(0.03)		

RI exp.—experimental index on non-polar column; The main volatiles and table summary are marked in bold.

**Table 2:** Enantiomer ratio (%) and enantiomeric excess (*ee*) of the main *A. concolor* seed and cone monoterpenes.

	*Abies concolor*	
Enantiomer	Seed	Cone
(*S*)-(−)-Limonene (%)	97.1	84.1
(*R*)-(+)-Limonene (%)	2.9	15.9
ee	94.2	68.2
(1*S*,5*S*)-(−)-α-Pinene (%)	87.0	95.8
(1*R*,5*R*)-(+)-α-Pinene (%)	13.0	4.2
ee	74.0	91.6
(1*S*,5*S*)-(−)-β-Pinene (%)	10.1	41.2
(1*R*,5*R*)-(+)-β-Pinene (%)	89.9	58.8
ee	79.8	17.6
(*S*)-(−)-Camphene (%)	-	94.2
(*R*)-(+)-Camphene (%)	-	5.8
ee	-	88.4

**Table 3:** Antimicrobial activities (MIC μL/mL) of *A. concolor* seed and cone essential oils and activities of thymol, as a positive control.

Essential Oil	Minimal Inhibitory Concentration MIC (μL/mL)				
	*Staphylococcus aureus* ATCC 43300	*Enterococcus faecalis* ATCC 51299	*Enterococcus faecium* ATCC 35667	*Escherichia coli* ATCC 25922	*Klebsiella pneumoniae* ATCC 700603
Seed	30 ± 1.4	26 ± 2.1	30 ± 3.5	26 ± 0.9	28 ± 1.8
Cone	30 ± 2.6	26 ± 0.7	26 ± 2.8	30 ± 2.2	28 ± 1.3
Thymol	2.5	0.13	0.39	0.63	0.76
